# A Study of the Phosphorylcholine Polymer Coating of a Polymethylpentene Hollow Fiber Membrane

**DOI:** 10.3390/polym15132881

**Published:** 2023-06-29

**Authors:** Feihua Ye, Zhisheng Chen, Chunsheng Li, Junhua Chen, Guobin Yi

**Affiliations:** 1 School of Environmental and Chemical Engineering, Zhaoqing University, Zhaoqing 526061, China; yefeihua@zqu.edu.cn (F.Y.); 15089670136@163.com (Z.C.); lichunsheng@zqu.edu.cn (C.L.); 2 Guangdong Provincial Key Laboratory of Environmental Health and Land Resource, Zhaoqing University, Zhaoqing 526061, China; 3 School of Chemical Engineering and Light Industry, Guangdong University of Technology, Guangzhou 510006, China

**Keywords:** phosphorylcholine, polymer, thermal decomposition, hollow fiber membrane, surface modification

## Abstract

A phosphorylcholine polymer (poly(MPC–co–BMA–co–TSMA), PMBT) was prepared by free radical polymerization and coated on the surface of the polymethylpentene hollow fiber membrane (PMP–HFM). ATR–FTIR and SEM analyses showed that the PMBT polymer containing phosphorylcholine groups was uniformly coated on the surface of the PMP–HFM. Thermogravimetric analysis showed that the PMBT had the best stability when the molar percentage of MPC monomer in the polymer was 35%. The swelling test and static contact angle test indicated that the coating had excellent hydrophilic properties. The fluorescence test results showed that the coating could resist dissolution with 90% (*v/v*%) ethanol solution and 1% (*w/v*%) SDS solution. The PMBT coating was shown to be able to decrease platelet adherence to the surface of the hollow fiber membrane, and lower the risk of blood clotting; it had good blood compatibility in tests of whole blood contact and platelet adhesion. These results show that the PMBT polymer may be coated on the surface of the PMP–HFM, and is helpful for improving the blood compatibility of membrane oxygenation.

## 1. Introduction

During COVID-19, membrane oxygenators achieved remarkable success in the life support of patients with pulmonary failure [1]. The core component of a membrane oxygenator is the hollow fiber membrane (HFM) material, the performance of which directly determines the performance of the oxygenator. Commonly used hollow fiber membrane materials are polypropylene (PP–HFM), cellulose acetate (CA–HFM), polyvinylidene fluoride (PVDF–HFM), and polymethylpentene (PMP–HFM) [2,3]. PP–HFM is the most widely used membrane material in clinical practice; however, directly prepared PP–HFM suffers problems such as a short life span and a tendency to fracture. CA–HFM has the characteristics of low cost and biodegradability, but it is susceptible to microbial corrosion and chemical attacks during utilization. PVDF–HFM has excellent oxidation resistance and mechanical strength, but its air permeability is average. PMP–HFM is a new type of membrane material that has been developed recently and has received a lot of attention because of its strong thermal stability, high mechanical strength, good air permeability, and chemical resistance [4]. However, no matter which hollow fiber membrane material is used, the surface is prone to protein adsorption, platelet adhesion, and thrombus deposition after prolonged contact with blood, leading to clinical adverse effects such as blood damage and inflammation, which often do not meet the requirements of prolonged medical treatment [5,6]. Therefore, the modification of membrane material surfaces via physical or chemical methods, in order to prepare hollow fiber membrane composites with excellent hemocompatibility, is currently a hot research topic.

Numerous efforts have been made to improve the blood compatibility on the surface of hollow fiber membrane materials, among which the construction of blood–compatible bionic coatings on the surface of fiber membranes is one of the most attractive solutions [7]. Using polymers containing phosphorylcholine (PC) groups to construct coatings on the surface of fibrous membranes can help to form amphiphilic ionic surfaces that mimic the cell membrane structure and can significantly improve the blood compatibility on the surface of fibrous membrane materials; 2-formyloxyethyl phosphorylcholine (MPC) is considered to be one of the most suitable monomers for the synthesis of phosphorylcholine polymers [8,9,10]. Phosphorylcholine polymer coatings have promising applications in extracorporeal circulation devices such as membrane oxygenators, stents, and blood purification devices [11,12,13].

Ishihara et al. [14,15,16] reported that the CA–HFM was coated with a copolymer (poly (MPC–co–BMA), PMB) to improve its performance with respect to solute and water permeability. The application of modified fiber membranes in blood purification devices and liver–assisted bioreactors has also been studied. Myers et al. [17] modified the surface of PP–HFM with poly(MPC–co–dodecyl methacrylate) and formed a polymer coating that could significantly reduce the blood circulation process of platelet activation. Teotia et al. [18] studied the in situ application of a phosphorylcholine polymer coating on PS–HFM to improve the blood compatibility of the membrane surface. Nishigochi et al. [19] coated the PMB polymer on PVDF–HFM to form a PMB coating, and found that the modified hollow fiber membrane could resist the adsorption of bovine serum protein. Wang et al. [20] used a dip–coating method to coat PMBT polymer on PP–HFM, and showed that the coating improved the hemocompatibility of the fiber membrane without hindering the gas exchange performance of the hollow fiber membrane. The authors of the paper coated poly(MPC–co–LMA–co–TSMA), PMLT) on PMP–HFM and found that the PMLT coating also showed a positive effect, improving the hemocompatibility of the fibrous membrane surface [21]. In addition, on the basis of earlier research, we found some new interesting phenomena. When different hydrophobic monomers, such as lauryl methacrylate (LMA) or *N*–butyl methacrylate (BMA), are used in the preparation of phosphorylcholine polymers, the differences in the hydrophobicity, chain length, and rigidity of the different monomers may lead to different bonding and contact angles between the polymer and the hollow fiber membrane surface, which in turn can affect the other related properties of the polymer coating. For this reason, we plan to further investigate the effect of phosphorylcholine polymers synthesized with different hydrophobic monomers on the modification of PMP–HFM.

Herein, we synthesized a phosphorylcholine polymer (poly(MPC–co–BMA–co–TSMA), PMBT) via a free radical polymer reaction, and coated the polymer on the surface of PMP–HFM to successfully prepare hollow fiber membrane composites with a PMBT coating on the surface. The morphology, thermal stability, hydrophilic properties, and chemical stability of the composite surface coating were tested via ATR–FTIR, SEM, TGA, contact angle, and fluorescence microscopy, respectively. Further, the blood compatibility of the coatings was analyzed using a platelet adhesion test and whole blood contact test. The results of the study can be used to prepare PMP–HFM bionic polymer coatings to improve the hemocompatibility of membrane oxygenators.

## 2. Experimental

### 2.1. Materials

2-Methacryloxyethyl phosphorylcholine (MPC, 97%) was purchased from Sigma–Aldrich, Sigma–Aldrich Trading Co., Ltd., Shanghai, China. *N*–butyl methacrylate (BMA, 99%), 3–(Trimethoxysilyl)propyl methacrylate (TSMA, 97%) and rhodamine 6G were supplied by Shanghai Aladdin Biochemical Technology Co., Ltd., Shanghai, China. The 2, 2′–Azoisobutyronitrile (AIBN, 99%) was recrystallized from methanol before being used. Platelet–rich plasma (PRP) was prepared using whole pig blood provided by Shanghai Yudo Biotechnology Co., Ltd., Shanghai, China. A polymethylpentene hollow fiber membrane (PMP–HFM, inner diameter 200 μm) was purchased from 3M Deutschland Gmbh, Seefeld, Germany. The sodium dodecyl sulfate (SDS), triethylamine (TEA, 99%), and other reagents were of analytical grade.

### 2.2. Synthesis of PMBT Polymer

**Figure 1:**
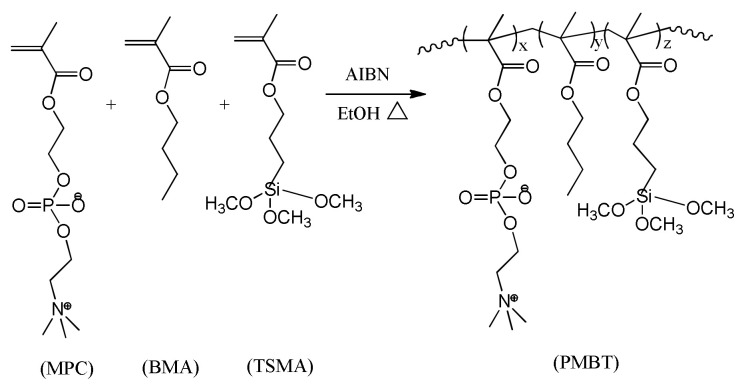
Synthesis route of the PMBT polymer.

The PMBT polymer was created via free radical polymerization, and the process of its making is shown in Figure 1 and Table 1. The necessary quantities of MPC, BMA, TSMA, and AIBN were weighed and then mixed together in ethanol, before being put into a funnel under constant pressure. In order to remove oxygen, nitrogen was bubbled into the reaction flask containing the solvent ethanol for 30 min. The flask was then heated to 83 °C. Drop by drop, the mixed reaction solution was added. The nitrogen gas flow was stopped once the dropwise addition was halted. After 24 h, the reaction was allowed to proceed before the heat source was cut off. The warm product solution was poured into a large amount of petroleum ether, and the polymer was precipitated while removing unreacted monomers to obtain a white flocculent precipitate. The precipitate was dissolved in absolute ethanol and precipitated again with petroleum ether. To produce a pure PMBT polymer, this procedure was performed three times. Finally, after being vacuum–dried at room temperature for 24 h, the polymer was then dissolved in a methanol solution (9:1 methanol to water volume ratio) to create a PMBT polymer solution with a specific concentration.

### 2.3. Preparation of the PMBT Coating

Coating [18,19,20,21,22], blending [23,24], and self–assembly [25,26] can be used to modify the surface of hollow fiber membranes with phosphorylcholine polymers. Among them, the coating method is widely used because of its convenience and simplicity. In this study, a coating method was used to prepare PMBT polymer coatings on the PMP–HFM of membrane oxygenators. The specific operation is as follows. The PMP–HFM was cut to a size of 2 cm × 2 cm, washed with ethanol and deionized water, respectively, and then dried under a vacuum at room temperature for 24 h. The clean PMP–HFM was immersed into the PMBT solution at room temperature for a certain time. Then, the coated PMBT film was crosslinked in an environment of 10% (*v/v*%) triethylamine aqueous solution for at least three days after the PMP–HFM was removed from the solution. Finally, the cross–linked phospholipid polymer surface was then completely washed with deionized water before being vacuum–dried at 30 °C.

### 2.4. Polymer Coating Analysis

The bare and PMBT35–coated PMP–HFM were characterized using ATR–FTIR via an IS 50R Fourier transform infrared (FTIR) spectrometer (Thermo Fisher Scientific, Waltham, MA, USA). The surface coating morphology of PMP–HFM was observed using an SU 8220 field emission scanning electron microscope (Hitachi, Tokyo, Japan).

### 2.5. Thermal Stability Test

The PMBT polymer thermal weight loss was tested using a TGA analyzer (Netzsch, Serb, Germany). The sample weight was about 6 mg, the heating rate was 20 °C/min, and the nitrogen protection was carried out. The test range was 25~800 °C.

### 2.6. Swelling Degree Test

The dried PMBT polymer was weighed and placed in distilled water for soaking, and the water on the surface was quickly blotted out gently with filter paper after being removed at certain time intervals, and the mass was weighed and recorded. In this way, the water content (*WC*) of the polymer was measured with the changing pattern of dissolution time. Each sample was tested three times, and the *WC* was calculated as follows.

(1) $$\mathrm{WC}=\frac{m_{t}-m_{0}}{m_{t}}\times 100\%$$

In the equation, *t* is the dissolution time of PMBT polymer in water, $m_{t}$ is the mass of PMBT polymer at the moment of dissolution *t*, and $m_{0}$ is the mass of dry PMBT polymer before dissolution.

### 2.7. Static Contact Angle Measurements

The water contact angle of the bare and PMBT35–coated PMP–HFM surfaces was measured using an OCA 100 optical contact angle-measuring instrument (DataPhysics, Filderstadt, Germany). To verify reproducibility, a minimum of ten samples were examined in each instance. In addition, the PMBT–coated PMP–HFM was immersed in 90% (*v/v*%) ethanol solution for 12 h or 1% (*w/v*%) SDS solution for 30 min, removed, and vacuum dried at 30 °C for 24 h. The static contact angle of the PMP–HFM surface was tested before and after treatment. The hydrophilic properties and stability of the PMBT coating were analyzed by comparing the change in the contact angle after ethanol soaking.

### 2.8. Fluorescence Properties of the Polymer Coating

The bare and PMBT35–coated PMP–HFM were submerged in a 200 ppm rhodamine 6 G solution in water for 30 s before undergoing two cycles of washing in clear water. Before examining with a 1X73 inverted fluorescence microscope (Olympus, Tokyo, Japan), the PMP–HFM was allowed to dry for 4 h in the open. To enable precise positioning, the PMP–HFM was viewed under normal lighting. After that, the microscope was set to fluorescence mode. The exposure time was 10 ms. The fluorescence excitation wavelength was 530~550 nm.

To further investigate the stability of the PMBT polymer coatings in ethanol and SDS solutions, the coated PMP–HFM was immersed in 90% (*v/v*%) ethanol solution for 12 h or 1% (*w/v*%) SDS solution for 30 min. The PMP–HFM was removed and stained with rhodamine 6 G solution, and the fluorescence performance of the coating was observed after treatment with a fluorescence microscope.

### 2.9. Platelet Adhesion

Pigs’ citrated whole blood was centrifuged at 1000 RPM for 10 min to create platelet–rich plasma (PRP). The bare and PMBT35 coated PMP–HFM were immersed in 0.01 mol/L PBS solution at pH 7.4 for 2 h until swelling equilibrium was reached. To get rid of the weak adhering platelets, the PMP–HFM was rinsed five times with PBS after being submerged in PRP for 1 h at 37 °C. To immobilize the blood components on the PMP–HFM, it was then submerged in 2.5 wt% glutaraldehyde and maintained at 4 °C for 12 h. The PMP–HFM was then freeze-dried at −50 °C after being repeatedly washed with deionized water. Using SEM, the samples’ surface was examined.

### 2.10. Whole Blood Contact

PBS was used to equilibrate the surfaces of the bare and PMBT35–coated PMP–HFM for two hours at room temperature. The samples were then submerged in the same porcine whole blood that had been citrated, and to balance off the anticoagulant effects of the citrate, a small amount of CaCl_2_ solution (0.2 mol/L) was added to the solution. The PMP–HFM was extracted, washed with deionized water, and fixed with 2.5 wt% glutaraldehyde at 4 °C for 12 h, after shaking for 2 h at a frequency of 70 cycles/min at 37 °C. SEM was used to examine the samples’ surface.

## 3. Results and Discussion

### 3.1. PMBT Coating Analysis

**Figure 2:**
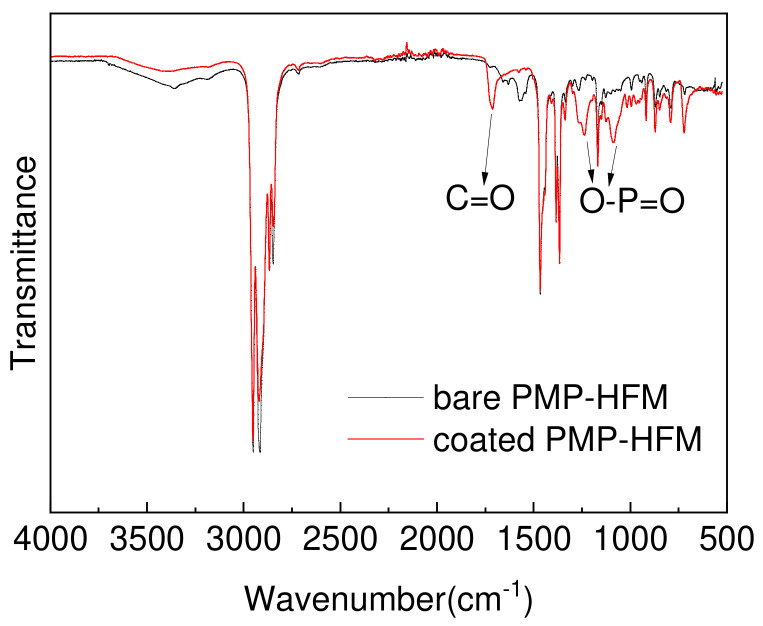
ATR-FTIR spectrum of bare and PMBT35-coated PMP-HFM.

It can be seen from Figure 2 that compared with the bare PMP–HFM, the PMBT35–coated PMP–HFM exhibits C=O absorption peaks at 1727 cm^−1^, and O–P=O absorption peaks at 1240 cm^−1^ and 1080 cm^−1^. As a result of infrared analysis, the surface of PMP–HFM has phosphorylcholine groups after being treated with PMBT solution.

**Figure 3:**
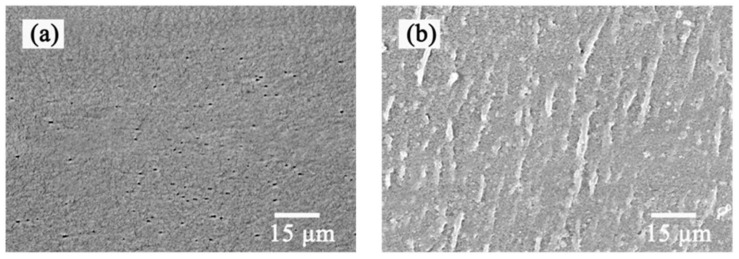
SEM images of bare (**a**) and PMBT35-coated (**b**) PMP-HFM.

Scanning electron microscope was used to observe the surface morphology changes of the PMBT35–coated PMP–HFM. The surface of bare PMP–HFM (Figure 3a) is significantly different from coated PMP–HFM (Figure 3b); the coated PMP–HFM surface is attached with a uniform PMBT polymer coating. When the polymer PMBT solution comes into contact with the PMP–HFM surface, the hydrophobic unit BMA in the polymer interacts with the fibrous membrane, which also has a hydrophobic surface, allowing the polymer to quickly adhere to the surface of the fibrous membrane via physical action. In addition, the cross–linkable unit TSMA in the polymer can form a network structure that further firmly encapsulates the PMBT polymer on the surface of the hollow fiber membrane, thus forming a stable PMBT coating.

### 3.2. Thermal Stability Analysis of PMBT Polymer

**Figure 4:**
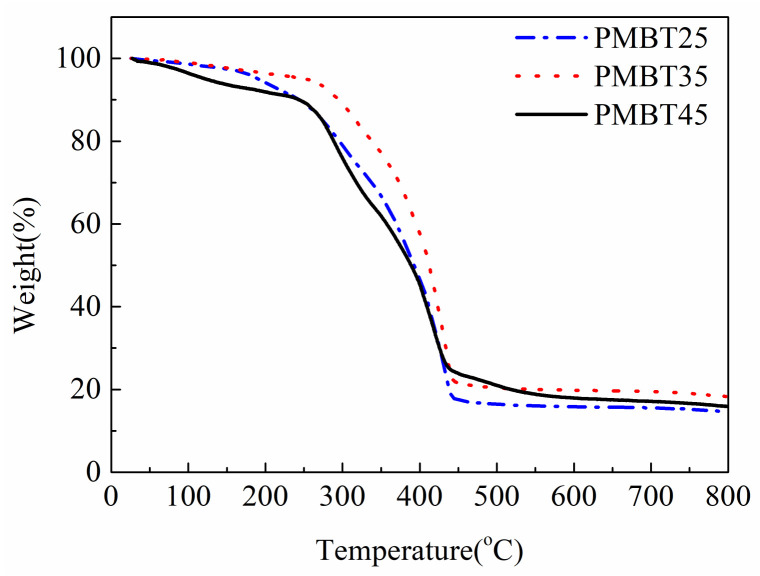
TGA curves of PMBT polymers.

Thermogravimetric analysis was used to test the thermal decomposition stability of PMBT polymer. T_(5%)_ is the temperature corresponding to the polymer mass loss of 5%, representing the initial decomposition temperature of the polymer, which can be used to judge the thermal stability of the polymer with different MPC contents. Combined with Figure 4 and Table 2, it can be seen that with the increase in the amount of MPC, the thermal decomposition temperature of the PMBT polymer increases first and then decreases, among which the initial decomposition temperature of the PMBT35 polymer is the highest (252 °C) and has the best stability.

The thermal decomposition of the polymer is mainly related to chemical bond energy and molecular structure. The MPC unit of PMBT polymer contains a bond energy of C–N (305 kJ/mol), which is lower than that of C–C (332 kJ/mol) and Si–O (460 kJ/mol), and the fracture decomposition occurs first in the process of thermal decomposition, resulting in mass loss of the polymer. In addition, the −OH produced by the hydrolysis of the TSMA unit in the polymer will form an intramolecular hydrogen bond with the O atom in the MPC unit, thus enhancing the thermal stability of the polymer. Therefore, when the amount of MPC is initially increased, the intramolecular hydrogen bond in the polymer increases the thermal decomposition temperature. When the amount of MPC reached a certain level, a large number of C–N bonds began to break in the process of polymer thermal decomposition, the mass loss of the polymer accelerated, and the thermal decomposition temperature decreased.

### 3.3. Swelling Degree Analysis of PMBT Polymer

**Figure 5:**
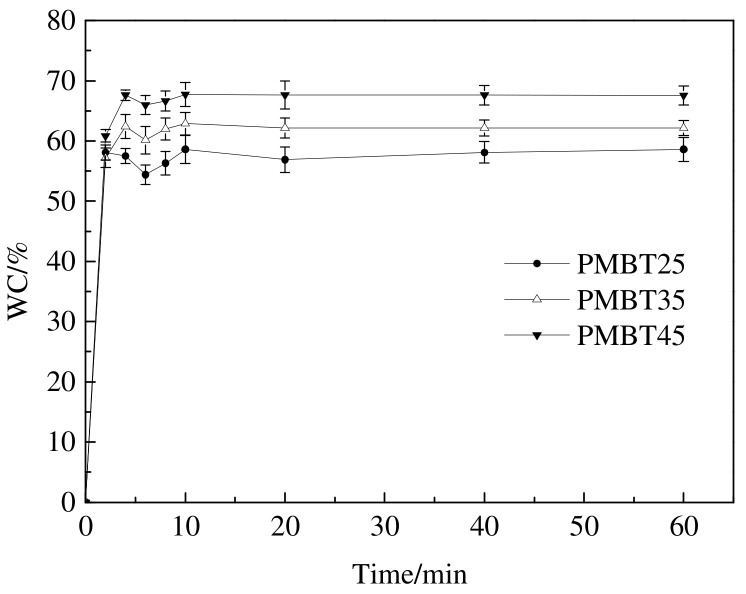
The relationship between *WC* and soaking time of PMBT polymers.

The study of the swelling behavior of PMBT polymer in water and the presence of water in the polymer is important for determining the hydrophilic properties and hemocompatibility of the polymer. As seen in Figure 5, the PMBT polymer rapidly absorbs water and reaches saturation within a few minutes. Subsequently, the water content within the PMBT polymer decreases slightly, and this particular phenomenon of water content rising and then falling during the swelling process is called over–swelling. The possible reason for this phenomenon is that the phosphorylcholine group (PC) contained in the PMBT polymer has a strong hydrophilic ability, which makes the polymer absorb water too quickly at the beginning of the swelling, meaning the molecular chain segments do not have time to spring back and a hysteresis effect occurs. In addition, the figure shows that the swelling of the PMBT polymer increases as the MPC concentration rises. This is due to the fact that each PC group can bind 12 water molecules, and the higher the MPC content, the more water molecules are bound to the PMBT polymer, which leads to a greater swelling of the polymer.

### 3.4. Static Contact Angle Analysis

Contact angle is an effective method used to observe the structure of the polymer surface and its changes, and a convenient means to measure the wetting degree of the polymer surface liquid. As seen from Table 3, the surface of bare PMP–HFM is hydrophobic. After coating with the PMBT polymer solution, the surface of PMP–HFM became hydrophilic. The hydrophilic surface was beneficial for improving the compatibility of the hollow fiber membrane with blood.

Furthermore, the coated PMP–HFM was immersed in 90% (*v/v*%) ethanol solution for 12 h or 1% (*w/v*%) SDS solution for 30 min, and the static contact angle on the surface of the PMP–HFM was tested. It was found that the contact angle was almost unchanged. This result shows that the PMBT coating can stably adhere to the surface of PMP–HFM. The stability of the PMBT coating was related to the introduction of the TSMA monomer during polymer synthesis. When the PMBT–coated PMP–HFM was immersed in the aqueous solution of triethylamine (TEA), the coating component, TSMA, first hydrolyzed to form a hydroxyl group attached to silicon. This hydroxyl group was very active and dehydrated with other hydroxyl groups to form ether bonds and cross–linking. The network structure allowed the PMBT polymer to tightly coat the surface of the PMP–HFM to form a stable polymer coating.

### 3.5. Analysis of the Fluorescence Properties of the Coatings

**Figure 6:**
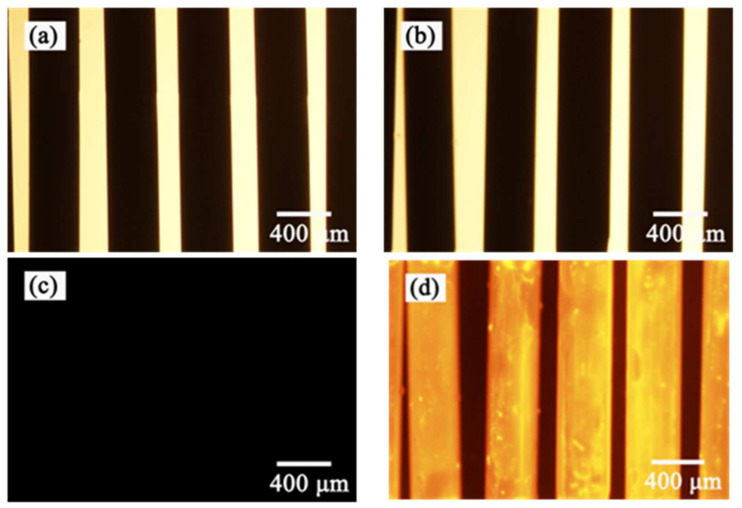

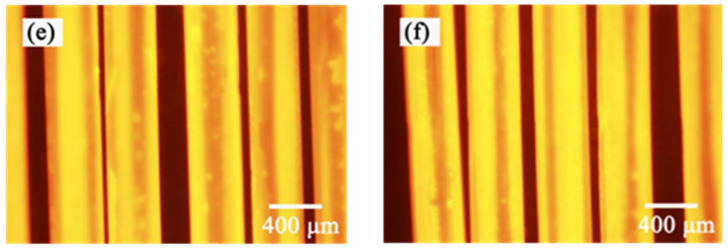
Microscope spectrum of bare and PMBT35-coated PMP-HFM. Underlit microscope images of bare (**a**) and coated (**b**) PMP-HFM; (**c**,**d**) are the same images viewed in fluorescence mode; (**e**,**f**) are fluorescence images of coated PMP-HFM after treatment with 90% (*v/v*%) ethanol solution or 1% (*w/v*%) SDS solution.

Rhodamine 6 G could interact with the PC group in the phosphorylcholine polymer and emit fluorescence under the action of specific excitation light [27]. Observing the fluorescence characteristics of the coating could be one of the methods used to evaluate the continuity and stability of the surface coating of the hollow fiber membranes [28]. Figure 6 shows a microscope image of bare (Figure 6a) and coated (Figure 6b) PMP–HFM under visible light. Because the PMP–HFM material was white and opaque, it appeared as a black tube under an inverted fluorescence microscope. After switching to fluorescence mode, the bare PMP–HFM (Figure 6c) had no fluorescence emission, showing a dark image, while the coated PMP–HFM (Figure 6d) emitted bright orange–yellow fluorescence, and the hollow fiber membrane could be clearly seen. Through continuous observation of the moving stage, it was found that the entire hollow fiber membrane surface emits fluorescent light, indicating that the PMP–HFM was uniformly covered by the PMBT polymer coating.

To further investigate the stability of the coating, the coated PMP–HFM was either immersed in 90% (*v/v*%) ethanol solution (Figure 6e) and left for 12 h, or was immersed in 1% (*w/v*%) SDS solution (Figure 6f) with 30 min of ultrasonic treatment. After drying, the sample underwent a dyeing treatment, and then the fluorescent effect of the coating on the surface of the hollow fiber membrane was observed. The results show that the fluorescence characteristics of the PMBT polymer coating on the surface of the PMP–HFM after treatment are still obvious. This result shows that the PMBT coating is stable in its adherence to the PMP–HFM, and can withstand ethanol or SDS solution dissolution.

### 3.6. Biocompatibility Evaluation

**Figure 7:**
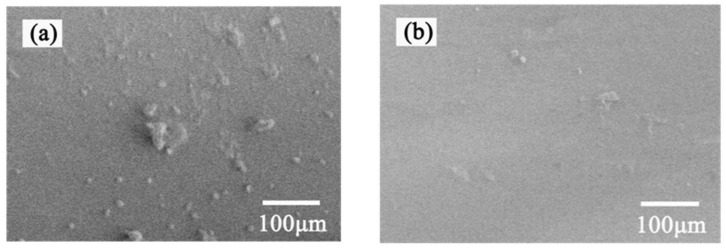
SEM images of platelet adhesion on the bare (**a**) and PMBT35-coated (**b**) PMP-HFM.

**Figure 8:**
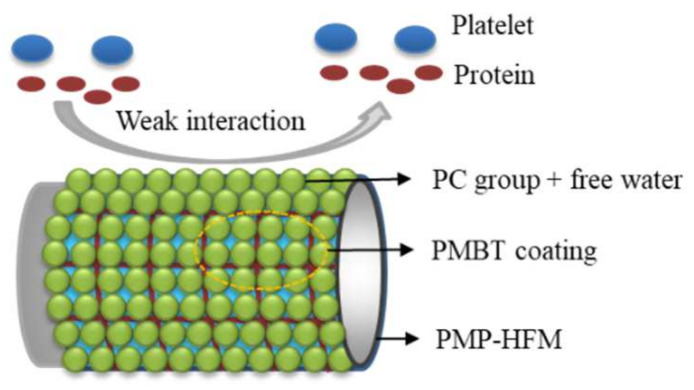
Schematic diagram of platelet and protein interaction on the coated PMP-HFM surfaces.

The adhesion and activation of platelets on the surface of medical materials is an important factor leading to coagulation. Therefore, studying the adhesion behavior of platelets on the surface of materials is an important method to evaluate the anticoagulant properties and blood compatibility of materials. It can be seen from Figure 7 that after the platelet adhesion experiment, there were many platelets adhered to the surface of the bare PMP–HFM (Figure 7a), and some of the platelets adhered thereon were aggregated and deformed. In contrast, the surface platelet adhesion of the PMP–HFM (Figure 7b) with the PMBT coating was significantly reduced, indicating that the PMBT coating can improve the blood compatibility of the PMP–HFM surface. The PMBT coating has a structure that mimics cell membranes. On the one hand, when in contact with blood, the phospholipids in blood will preferentially adsorb on the coating surface over proteins and platelets, forming a bionic surface and reducing protein adsorption; on the other hand, the polymer surface maintains a high free water content, and the contact between proteins and the polymer surface is reversible, which does not cause protein conformation changes and avoids protein adsorption. The biocompatibility of the PMBT35–coated PMP–HFM is shown schematically in Figure 8.

**Figure 9:**
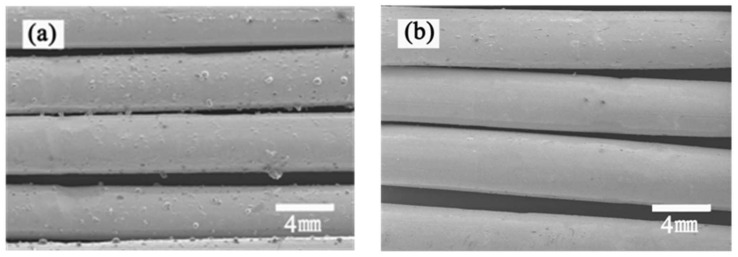
SEM images of whole blood contact on the bare (**a**) and PMBT35-coated (**b**) PMP-HFM.

The whole blood contact test results showed that after the hollow fiber membrane was immersed in the blood for two hours, the surface of the bare PMP–HFM (Figure 9a) was adhered to by more thrombi, while the surface of the coated PMP–HFM (Figure 9b) was very clean, with no visible thrombus adhesion. The PMBT coating has an excellent antithrombotic effect; as a result of the amphiphilic phospholipid polymer coating’s ability to mimic the outer membrane of cell, it exhibits significant resistance to platelet attachment and protein absorption. Therefore, the blood compatibility of the material surface can be significantly improved by coating the surface of the PMP–HFM with PMBT coating.

## 4. Conclusions

We have successfully synthesized a phosphorylcholine polymer PMBT and applied it to the surface of PMP–HFM, uniformly, via a dip–coating method, to form a stable biocompatible coating. ATR–FTIR and SEM results confirmed that a PMBT coating was formed on the surface of PMP–HFM, and that the coating was uniformly encapsulated on the surface of the fiber membrane. The thermogravimetric test results showed that with the increase in the amount of MPC, the thermal decomposition temperature of the PMBT polymer increases first and then decreases, among which the initial decomposition temperature of PMBT35 polymer is the highest. The swelling test results indicate that the coating had excellent hydrophilic properties. Static contact angle and fluorescence microscopy results showed that the network–like structure formed in the polymer due to the introduction of the cross–linkable monomer TSMA enables the PMBT coating to remain stable in extreme environments, exhibiting good chemical stability. The results of platelet adhesion and whole blood contact experiments showed that the phosphorylcholine polymer PMBT could significantly improve the blood compatibility of PMP–HFM surface, thus prolonging the contact time between the hollow fiber membrane and blood, and providing a new solution idea for the clinical application of PMP–HFM. Encouragingly, the polymer coating is very simple to prepare, and the coating is uniform and stable; this is promising for application in the mass production of membrane oxygenators, which will create more blood–compatible membrane oxygenators with a longer service life.

## Figures and Tables

**Table 1:** Monomer feed ratio of PMBT polymer.

Sample	MPC (mol%)	BMA (mol%)	TSMA (mol%)
PMBT25	25	65	10
PMBT35	35	55	10
PMBT45	45	45	10

**Table 2:** TGA data of different MPC contents of PMBT polymers.

Samples	T_(5%)_/°C	T_(10%)_/°C	T_(50%)_/°C
PMBT25	191	245	393
PMBT35	252	295	412
PMBT45	123	244	389

**Table 3:** Static contact angles of the bare and PMBT35-coated PMP-HFM.

Samples	Bare PMP-HFM	PMBT35 Coated PMP-HFM	Treated with Ethanol Solution	Treated with SDS Solution
θ (deg)	106 ± 0.6 *	79 ± 0.8 *	77 ± 1.3 *	76 ± 1.1 *

* *p* < 0.001 vs. bare.

## Data Availability

The data presented in this study are available on request from the corresponding author.
